# Supplementary material for: Implementation costs and cost-effectiveness of ultraportable chest X-ray with artificial intelligence in active case finding for tuberculosis in Nigeria
Source: PLOS Digit Health. 2025 Jun 11;4(6):e0000894. doi: 10.1371/journal.pdig.0000894 (PMC12157241; doi:10.1371/journal.pdig.0000894)

S5. Cost per case diagnosed across different x-ray and AI equipment scenarios

Only algorithm utilizing CXR are presented here. There is no difference for symptoms-only algorithms because neither CXR nor AI are utilized in these algorithms.

Cost per TB Diagnosed – Abnormality ≥ 0.30 OR any symptom

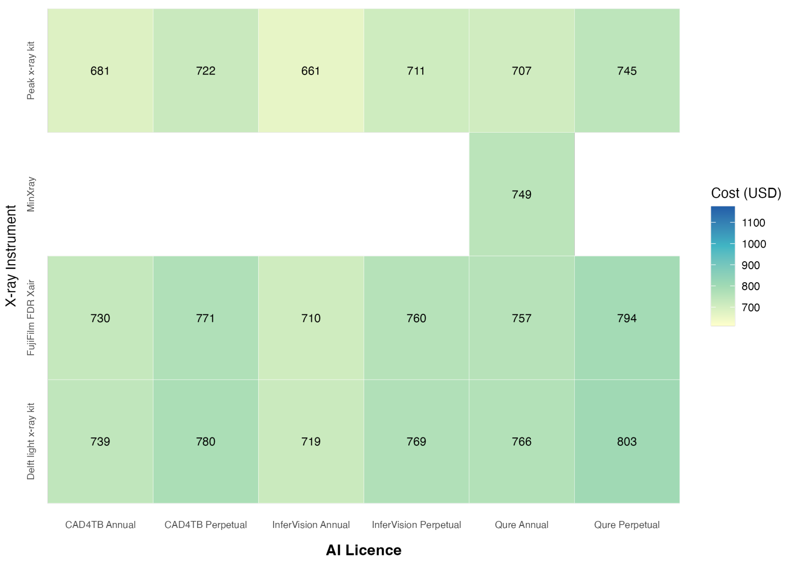

Cost per TB Diagnosed – Abnormality ≥ 0.30

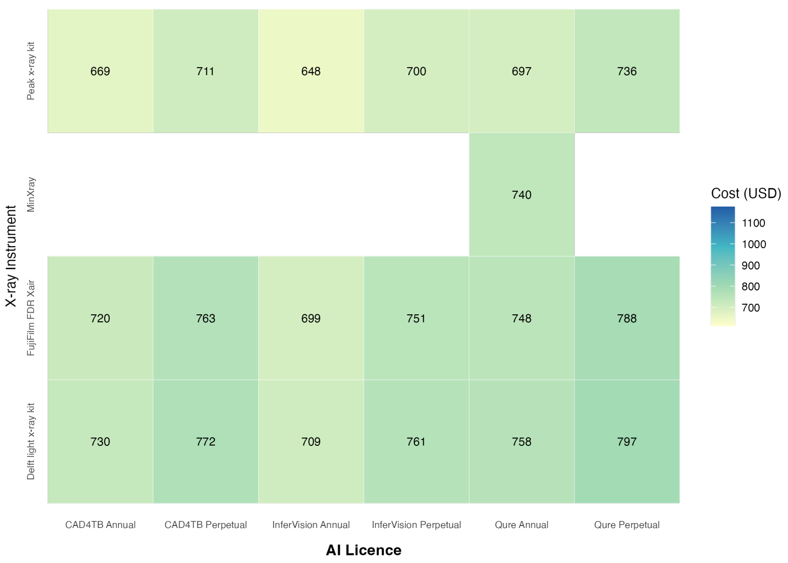

Cost per TB Diagnosed – Abnormality ≥ 0.50

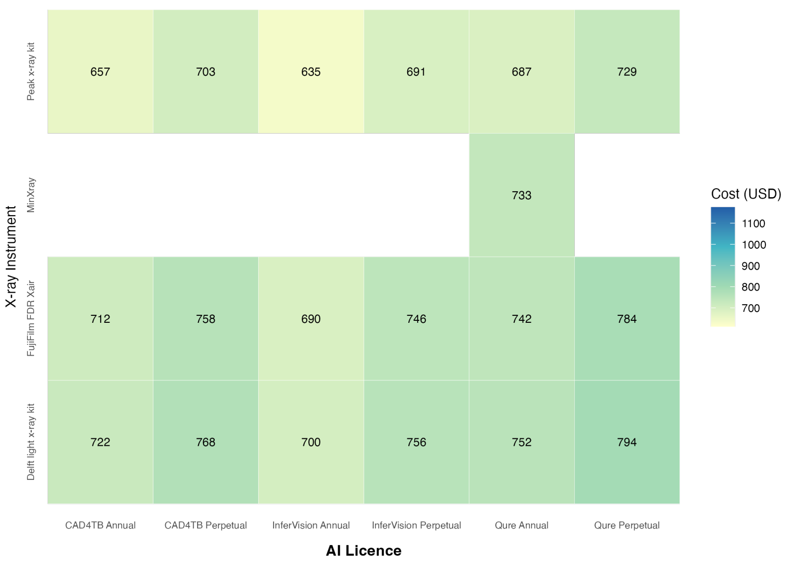

Supplement: S5 File — These are results of sensitivity analysis using cost data from the Global Drug Facility’s Diagnostics, Medical Devices & Other Health Products Catalog, March 2025. All scenarios include prices from this catalog for Xpert Ultra cartridge, X-ray and AI equipment. The base case uses actual prices for X-ray and AI equipment with current prices for Xpert Ultra cartridge. Ultra has replaced MTB/RIF, which is no longer available in the catalog. (PDF) [file pdig.0000894.s005.pdf]
