# Supplementary material for: Implementation costs and cost-effectiveness of ultraportable chest X-ray with artificial intelligence in active case finding for tuberculosis in Nigeria
Source: PLOS Digit Health. 2025 Jun 11;4(6):e0000894. doi: 10.1371/journal.pdig.0000894 (PMC12157241; doi:10.1371/journal.pdig.0000894)
Supplement: Attachment — Submitted filename: R1 response.docx [file pdig.0000894.s007.docx]

# Response to reviewers and editor

Thanks to the reviewers and editor for their constructive comments. In this revised version, we have addressed your feedback.

We have also revised the title of the paper to include TB in it. The new title is “Implementation costs and cost-effectiveness of ultraportable chest X-ray with artificial intelligence in active case finding **for tuberculosis** in ~~Northeast~~ Nigeria”

Additional Editor Comments

This is a very valuable contribution, thanks for submitting it. I have two main points that need to be addressed before publication:

1) The willingness to pay threshold based on GPD pre capita used here is usually employed with respect of one QALY gain. In your setting you have additional detected cases as the outcome. Please link the gain to QALY gains from the additional detection (and then use this in combination with the GDP) to get to a threshold or use another measure for social willingness to pay for one additional detected case.

Thank you for raising this point. We agree that GDP per capita as WTP threshold not the most appropriate WTP threshold for the cost per case detected outcome. It is not as straightforward to convert this to QALY gains, therefore we have chosen to remove the WTP threshold in both figures and text. We think the main results hold independently of that in showing the comparison between algorithms – combining CXR-AI with symptoms for screening is preferable than symptoms only screening in terms of cost per case and missed cases.

2) The license costs you use in your estimation differ widely from the ones reported in the discussion (based on reference 32). Please add a cost-effectiveness analysis taking into account the possible real costs for full implementation.

We have added a sensitivity analysis using several different x-ray and AI equipment available in the current GDF catalog, along with current pricing of Xpert cartridge cost (March 2025; reference modified in revised manuscript). These are compared with base case for all five algorithms. The figures are presented in supplementary file S5. The main finding is that cheaper x-ray units make a larger difference than AI license (annual slightly lower than perpetual license in this scenario with an intervention over short duration.)

Minor Points:

- I agree with reviewer 2 that electricity sounds like costs that should be in there.

We have addressed this. Please see our response to the reviewer below.

- I could not replicate the results based on the Rmd files provided in GitHub. Please add a ReadMe file of how the codes need to be executed in which order and where which datasets from where need to be placed to get the results. Idealy there is one MAIN File that runs everything. You could also think about automating the flow with makefiles. You might also find that updating from R-Markdown to Quarto gives you more power.

Thank you for the suggestion on moving to Quarto. I plan to start new projects in Quarto. For now, we have added the data files and all other information to the repository. The README has instructions to execute the code using a script to batch run all the Rmd analyses files.

# Reviewer #1 comments

Reviewer #1: A worthwhile and timely article. I acknowledge the time and effort the authors have invested in this research.

While the authors employed a health system perspective for the analysis, a societal perspective would have been preferable to capture all externalities, including those associated with environmental costs of implementing the program.

Thank you for the encouragement. We agree with you on the need for using a societal perspective for a full cost-effectiveness analysis. We write in the discussion that “future studies should undertake prospective costing using micro-costing approaches over longer intervention duration in different population settings while considering both health system and societal perspectives.”

# Reviewer #2 comments

Reviewer #2: This study discusses the cost-effectiveness and implementation costs of using ultraportable chest X-rays (CXR) combined with artificial intelligence (AI) in active case finding (ACF) for tuberculosis (TB) in Northeast Nigeria. The study involved people 15 years and older in Northeast Nigeria. The authors compared five screening scenarios, including symptom-based methods and CXR-AI approaches, to identify the most efficient strategy for detecting TB cases. The findings demonstrated that combining CXR and AI with symptom screening detects more cases at a lower cost per case diagnosed.

Overall, the paper contributes to understanding the cost implications and effectiveness of adopting CXR and AI for TB screening in ACF.

The paper is highly relevant as it addresses a critical global health issue: improving TB detection through the adoption of CXR and AI. The authors have also clearly explained the methodology and provided the dataset and code, which aids the reproducibility of the findings.

Thank you for the appreciation. We have made changes based on your comments.

I didn't see the cost of electricity in the cost parameter. Was electricity not a factor in the experiment? If it was, the authors should clarify how it was accounted for.

We appreciate your careful observation. The cost of electricity is included in “fuel cost accessing screening sites.” The devices (battery-powered x-ray and laptop) were charged and run on power from the vehicle. We have included this now in legend of table 1 and supplementary table 1: “This cost item also includes the cost of charging the battery-powered x-ray and laptop devices using power from the vehicle.”

As for other electricity costs like running the lab, we did not include that and clearly lay it out in the methods: “We only estimated additional cost of implementing the ACF intervention on top of a functional TB program. Therefore, we did not include the cost of running a basic TB program (management, notification, treatment initiation), cost of lab facilities (apart from cost of GeneXpert equipment, cartridges, and laboratory support incentives), and long-term operational costs like equipment maintenance.”

While this study has numerous strengths, there are a few concerns: The study is more focused on resource-limited regions and is limited in generalizability to other areas due to the peculiarity of the studied region. Expanding the discussion on how these findings could be adapted to different populations or healthcare settings would be beneficial.

The authors discussed limitations briefly but could provide more detail on the challenges of replicating these findings in urban or higher-resource settings.

Thank you for highlighting this. We have made revisions in the text. Mainly, we have added the following in the discussion. “Our study was in a resource-limited setting with a unique population group which limits the generalizability to other contexts. ACF implementation is highly contextualized and needs customization compared to similar intervention at facility.[17] ACF is also often more expensive because of the need for extra effort in providing services to people with poorer access to health system. In replicating such an intervention in another setting like urban area or facility, TB prevalence, including that of asymptomatic TB, and service utilization will be important determining factors for cost efficiency.”

Also, there are minor grammatical errors, such as "promising developments tuberculosis (TB) active case finding" (which should be "promising developments in tuberculosis (TB) active case finding").

Thanks for your close reading. We have proofread the revised manuscript. We have also added minor clarifying details across the manuscript.
