# Supplementary material for: Implementation costs and cost-effectiveness of ultraportable chest X-ray with artificial intelligence in active case finding for tuberculosis in Nigeria
Source: PLOS Digit Health. 2025 Jun 11;4(6):e0000894. doi: 10.1371/journal.pdig.0000894 (PMC12157241; doi:10.1371/journal.pdig.0000894)
Supplement: Attachment — Submitted filename: R2 response.docx [file pdig.0000894.s008.docx]

# Response to editor

Thanks for the revision of the paper. One last minor adjustment: Can you please make make the sentence "Using CXR and AI had the same cost per case detected when clinically diagnosed cases constituted 30% of all TB diagnosed" a bit more clear in the Abstract and add this main message also to the Author Summary as well as the Discussion section.

Thank you for highlighting this. We have revised it and added it to the sections as suggested. It reads, “Using CXR and AI had lower cost per case detected than any symptom screening criteria when asymptomatic TB was higher than 30% of all bacteriologically- confirmed TB detected.”

From Figure 4 I would have thought that "when more than 30 percent of TB cases are asymptomatic, then the costs for one additional detected case is lower when CXR and AI are used compared to the baseline". Which means that if I suspect a lot of asymptomatic cases, then I should use CXR and AI, if most of the cases are symptomatic, it is not worth the extra effort. I feel like this is the important takaway here.

You are absolutely correct, and we agree with you. We have expanded this in the discussion: “Our analysis showed that using CXR and AI had lower cost per case detected than any symptom screening criteria when asymptomatic TB was higher than 30% of all bacteriologically-confirmed TB detected. (Fig 4) While the exact threshold will vary based on test costs, generally, CXR and AI will be a more cost efficient strategy in screening settings with higher proportions of asymptomatic TB.”

We have also changed subclinical TB to asymptomatic TB for consistency.
